# Empirical Evidence for Intraspecific Multiple Realization?

**DOI:** 10.3389/fpsyg.2020.01676

**Published:** 2020-07-24

**Authors:** Francesca Strappini, Marialuisa Martelli, Cesare Cozzo, Enrico di Pace

**Affiliations:** ^1^Neurobiology Department, Weizmann Institute of Science, Rehovot, Israel; ^2^Department of Psychology, Sapienza University of Rome, Rome, Italy; ^3^Department of Philosophy, Sapienza University of Rome, Rome, Italy

**Keywords:** multiple realizability, identity theory, visual integration, natural kinds, antireductionism, crowding, object recognition, functionalism

## Abstract

Despite the remarkable advances in behavioral and brain sciences over the last decades, the mind-body (brain) problem is still an open debate and one of the most intriguing questions for both cognitive neuroscience and philosophy of mind. Traditional approaches have conceived this problem in terms of a contrast between physicalist monism and Cartesian dualism. However, since the late sixties, the landscape of philosophical views on the problem has become more varied and complex. The *Multiple Realization Thesis* (MRT) claims that mental properties can be (or are) realized, and mental processes can be (or are) implemented by neural correlates of different kinds. Thus, MRT challenges the psychoneural type-identity theory and the corresponding reductionism. Many philosophers have acknowledged the *a priori* plausibility of MRT. However, the existence of empirical evidence in favor of intraspecific, human multiple realizations of mental processes and properties is still controversial. Here, we illustrate some cases that provide empirical evidence in support of MRT. Recently, it has been proposed that foveal agnosic vision, like peripheral vision, can be restored by increasing object parts’ spacing (Crutch and Warrington, 2007; Strappini et al., 2017b). Agnosic fovea and normal periphery are both limited by crowding, which impairs object recognition, and provides the signature of visual integration. Here, we define a psychological property of restored object identification, and we cross-reference the data of visually impaired patients with different etiologies. In particular, we compare the data of two stroke patients, two patients with posterior cortical atrophy, six cases of strabismic amblyopia, and one case with restored sight. We also compare these patients with unimpaired subjects tested in the periphery. We show that integration (i.e., restored recognition) seems to describe quite accurately the visual performance in all these cases. Whereas the patients have different etiologies and different neural correlates, the unimpaired subjects have no neural damage. Thus, similarity in the psychological property given the differences in the neural substrate can be interpreted in relation to MRT and provide evidence in its support. Finally, we will frame our contribution within the current debate concerning MRT providing new and compelling empirical evidence.

## Introduction

Understanding what the mind is, its nature, and how it relates to the physical matter, the brain, represents one of the most basic and powerful questions through all human history. Nevertheless, for both science and western philosophy, a definitive answer remained elusive. On the one hand, cognitive neuroscientists have tried to address the problem on an empirical basis by studying the brain mechanisms underlying the cognitive functions, such as visual perception, learning, memory, and so on. On the other hand, philosophers of the mind have approached the problem from a broader point of view and have raised questions on how the mind is related to the existence of the body and how does it fit into the natural world.

The mind–body relation as a “problem” can be traced back to seventeenth century French philosopher René Descartes who asked how the material body, which works according to the physical laws, can interact with the immaterial mind. However, it is widely held that the modern debate over the mind–body problem began only later in the 1950s when physicalism, the view that everything that exists has an ultimate physical nature, became the dominant metaphysical perspective. Inspired by physicalism, the British philosopher and psychologist Place (1956), the Austrian philosopher Feigl (1958), and the Australian Philosopher Jack Smart (1959) proposed a view that became popular as the “identity theory,” which claims that mental states are identical with physical states in the following sense: for each type of mental state M there is a (finitely specifiable) type of physical state P such that any individual x is in the state M if, and only if, x is in P. According to this view, the mind–body problem is solved by recognizing that any mental state is a neurophysiological state in the nervous system – rather than having its neural correlates. This view is sometimes called psychoneural “type-identity” theory because it is maintained that the relevant type of mental state is a type of neurophysiological/physical state. Moreover, the term “type” highlights that both the mental and neural states are intended to be a general class of events (types), such as the mental state of feeling pain, rather than specific, spatiotemporally individuated instances (“token”) of a certain type, such as the same feeling of pain experienced in different conditions and timing.

Although this new approach was in line with the optimistic mood about the role of the modern science of that time and set some basic and useful constraints for future debates on the mind–body problem, it was eventually short-lived. One of the biggest challenges for the identity theory was the new fundamental change in approaching the problem that arose in the philosophic scenario, called functionalism. In 1967, the American philosopher Hilary Putnam, with the paper “Psychological Predicates” and other works, proposed that a mental state is a functional or computational state. So, the mind–body problem was solved by considering the mind neither as a non-physical thing nor as a physical one, such as a neurophysiological state, but rather in relation to its functionality. Following the naive brain–computer analogy, two computers can compute the same task (function) yet have two different physical states or hardware. So, two nervous systems can perform the same mental task (function) yet having two different neurophysiological states. This argument, known as the *Multiple Realization Thesis* (MRT) implies that mental states can be implemented by different neural correlates. Although MRT originated from the theoretical framework of functionalism; nowadays, it is considered separate and independent. In its original conception, MRT was applied across species; Putnam suggested that one mental state, like feeling pain, is likely realized with non-identical neurophysiological states in different animal species, like reptiles, birds, and mollusks (Putnam, 1967). The argument is more or less the following: suppose that a token m is John’s being in pain at time t and that m coincides with the excitation of a neural C-fiber (type P); suppose further that m^∗^ is a mollusk’s being in pain at time t^∗^ and that m^∗∗^ is an extraterrestrial creature’s being in pain at time t^∗∗^: the corresponding physical states of the mollusk and of the extraterrestrial creature are of types P^∗^ and P^∗∗^, different from excitation of a C-fiber (i.e., different from P). The tokens m, m^∗^, and m^∗∗^ are all instances of the state M of being in pain, but they correspond to tokens of different physical types: this is what is meant by saying that the mental type M is multiply realized. The argument can be also formulated in terms of mental properties: x is in a state M if, and only if, x has the property of being in M: so, mental properties are multiply realized. The reader may object that this argument may seem plausible, but it has two shortcomings: it lacks sound empirical support, and it concerns different species (one of which is only imaginary).

With this thesis, Putnam suggested that there is not a constant and invariant identity relation between mental and physical states as the identity theory holds. It also challenged all the reductionist approaches that claim that the physical substrate of the mind is exclusively the nervous system.

A few years later, Fodor (1974) extended Putnam’s thesis to intraspecies cases. He argued that mental states can have multiple realizations in the nervous system of different individuals that belong to the same species or even in the same individual across different brain states over time. According to this thesis, for (at least some) types of mental states M, there is not a finitely specifiable physical (neural) state P, such that any individual x is in the state M if, and only if, x is in P, because M is multiply realized. In other words, every single token m of type M is a physical (neural) token p of some type, but there is not a finitely specifiable physical type P of which all p’s are tokens: different tokens of M correspond to tokens of different physical types.

Although many philosophers acknowledged the plausibility of the argument, clear empirical evidence in favor of intraspecific, human multiple realization of mental states and properties is still missing. On the one hand, findings from cognitive neuroscience have been used both to support and oppose MRT depending on the grain of analysis used (Bechtel and Mundale, 1999). On the other hand, the way that the question of multiple realizations of mental states and properties has been posed, influenced, at least in part, the kinds of answers that have been proposed (Aizawa and Gillett, 2009a).

In this paper, we intend to remedy these shortcomings by describing empirically tested evidence of human multiple realization of mental states. Several formulations of MRT have been proposed in the literature (e.g., Shapiro, 2004; Polger and Shapiro, 2016). To avoid misunderstandings, one must choose one precise definition of multiple realization. Here, we adopt the formulation proposed by Aizawa and Gillett (2009a;b).

We will present two partially independent cases of multiple realization of a similar psychological property. In both cases, we will capitalize on a well-studied phenomenon, visual crowding, whereby an object cannot be identified in peripheral vision if surrounded by closely spaced elements. Recognition is restored when objects are separated by a range that describes the size of the integration mechanisms responsible for recognition. Thus, our psychological property is defined as a function of a physical parameter in the input stimulus. Based on this clear-cut definition of the property, we can make predictions on when and how the property is realized.

## Multiple Realization of Crowded Objects Identification

### Multiple Realization Definition

In the last two decades, the concept of realization and multiple realization has become the focus of a more stringent analysis. One of the most influential accounts of multiple realization has been proposed by Aizawa and Gillett (2009a; b), stemming from their “dimensioned” framework for realization relations.

According to their multiple realization definition:

A property G is multiply realized if and only if:

(i) under condition $, an individual s has an instance of property G in virtue of the powers contributed by instances of properties/relations F_1_ − F_n_ to s, or s ’s constituents, but not vice versa;
(ii) under condition $^∗^ (which may or may not be identical to $), an individual s^∗^ (which may or may not be identical to s) has an instance of a property G in virtue of the powers contributed by instances of properties/relations F^∗^_1_ − F^∗^_m_ to s^∗^ or s^∗^ ’s constituents, but not vice versa;
(iii) F_1_ − F_n_ ≠ F^∗^_1_ − F^∗^_m_ and
(iv) under conditions $ and $^∗^, F_1_ − F_n_ and F^∗^_1_ − F^∗^_m_ are at the same scientific level of properties” (Aizawa and Gillett, 2009a, p. 188).

In this framework, “a property is individuated by the causal powers it potentially contributes to the individuals in which it is instantiated,” and the realizer contributes to the power of the property and not vice versa (Aizawa and Gillett, 2009a).

The underlying idea is the following. The realized property G belongs to a certain scientific level or if you prefer, a certain layer of scientifically investigated reality. In our case, G belongs to the psychological level. The realizers, on the other hand, belong to a different, “lower,” scientific level. There can be different levels: microphysical, molecular, cellular, etc. We have a realization of G if properties F_1_ − F_n_ at a lower level L determine G under a condition $ [this is what clause (i) states]. We have another, different, realization of G if different properties F^∗^_1_ − F^∗^_m_ at a level L^∗^ determine G under a condition $^∗^ [this is what clause (ii) states]. Obviously, to have multiple realization, F_1_ − F_n_ must be different from F^∗^_1_ − F^∗^_m_ [this is what clause (iii) states]. However, (i), (ii), and (iii) would be trivially true if the levels L and L^∗^ were different (e.g., the cellular level and the microphysical level). That is why, to have genuine multiple realization the level of realizers must be the same, that is, L = L^∗^, and clause (iv) must be fulfilled.

In the next paragraph, we will briefly discuss visual crowding to define our properties.

### Visual Crowding

**FIGURE 1:**
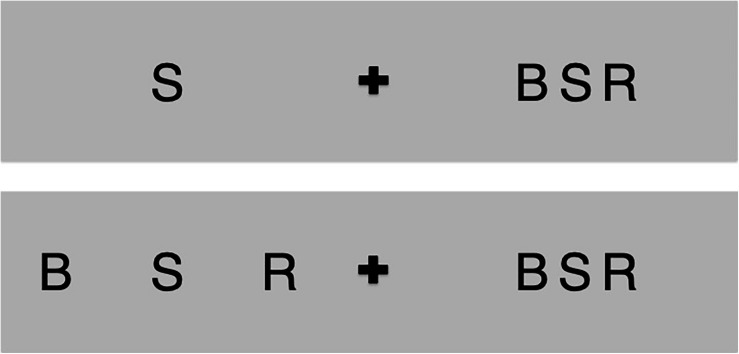
Try to identify the letter S while looking at the central plus in the upper panel. You will see that it is easy on the left and hard on the right. This is crowding: the recognition is hard on the right because the letter S is presented between flankers. Note that this difficulty is not due to acuity, as the targets have the same size. Now look at the plus in the lower panel; again, the recognition is easy on the left and hard on the right. You can escape crowding and restore recognition by increasing the flankers spacing up to a center-to-center distance greater than half of the target viewing eccentricity.

The entire chain of processing from sensation to object recognition is still partially underspecified. Feature detection, the process of filtering perceptually significant elementary units, like edges, is the first step of visual recognition, and it is considered a well-understood phenomenon (Hubel and Wiesel, 1965; Campbell and Robson, 1968; Graham and Nachmias, 1971). The visual system then binds or integrates the detected features to achieve an object representation that enables recognition. The nature of the feature integration process has long been debated. Recently, several studies have shown that a visual phenomenon called “crowding” could shed light on this feature integration process (Pelli et al., 2004; Pelli and Tillman, 2008; Whitney and Levi, 2011). When integration succeeds, the outcome is a correct object recognition; when it fails, we experience crowding, whereby the objects cannot be correctly identified. In this condition, the features mingle together and produce a jumble that is difficult to recognize. You can experience crowding yourself by looking at Figure 1. Crowding occurs when the object to identify is surrounded by nearby objects (like letters in words). Recognition is restored if the objects are spaced far enough apart to exceed the integration region (i.e., the area of the visual field in which features are integrated). Visual crowding is an essential bottleneck for object recognition and visual consciousness (crf. review by Levi, 2008). Crowding has pervasive effects in everyday life because most of the time, the majority of the visual scene is crowded, like words in a text.

Crowding is considered a mid-level phenomenon that impairs recognition while preserving detection (Pelli et al., 2004). It is operationally defined by psychophysical models that attempt to account for the computation that occurs in the integration region (the region of the visual field where the integration process is computed). These models of crowding suppose feature integration, pooling, source confusion (Treisman and Gelade, 1980; Treisman and Schmidt, 1982; Parkes et al., 2001; Chung et al., 2007; Nandy and Tjan, 2007; Levi, 2008), or a combination of all of these factors (Harrison and Bex, 2017). Pooling refers to compulsory averaging of some elementary feature characteristics, such as orientation, with loss of information about individual elements (Parkes et al., 2001). As for source confusion, this indicates the attribution of one of the object properties to a nearby object, for example, migration of simple oriented elements or color (Treisman and Schmidt, 1982; Nandy and Tjan, 2007).

One essential parameter that characterizes crowding, and that will be important for the definition of our mental property, is the *critical spacing*, the center-to-center spacing between the target object and the flankers needed for recognition (Bouma, 1970). This spacing is proportional to the eccentricity (angular distance from fixation) and independent of object size (Pelli et al., 2004). Specifically, when objects (such as letters or facial features) can be isolated from nearby elements by a critical spacing, features are correctly integrated, and recognition succeeds (Martelli et al., 2005; Chung et al., 2007; Grainger et al., 2010; Rosen et al., 2014; Herzog et al., 2015).

In general, the crowding range, the amount of spacing needed for recognition in any visual field position, defines the size of the integration regions. These regions tile up the entire visual field, but at each retinal location, there is a limit determined by the smallest integration region available at that location. If the smallest available region is too large to isolate the target from the flankers, crowding occurs, and the recognition is impaired. When the smallest integration region available matches the object size excluding the rest, recognition is possible. For this reason, the integration region has also been called *isolation field* to highlight the function of excluding everything that is outside it (Pelli et al., 2004; Martelli et al., 2005). Hereafter, we will use the term crowding and integration interchangeably, as crowding is a ubiquitous, by-product of feature integration.

Some particular types of neuropsychological patients have been reported to require an exceptionally large spacing to restore recognition in foveal vision. Visual crowding was first reported in the foveal vision of strabismic amblyopia and then in normal peripheral vision (Korte, 1923; Irvine, 1945). Recently, it has been shown that also foveal vision in patients with visual agnosia, posterior cortical atrophy (PCA), and visual deprivation is limited by crowding, like peripheral vision in normal subjects (Martelli et al., 2000; Crutch and Warrington, 2009; Strappini et al., 2017b). Surprisingly, these patients have very diverse lesions, all accidental, and sometimes, they do not have any lesion at all. However, they share the same visual behavioral pattern.

In the next paragraphs, we will formalize our evidence of multiple realization by considering two parallel accounts for MRT.

In the first study, we will set the condition requirements according to a stricter definition. The criteria isolate a psychological property characterized by identical input parameters across patients in foveal vision. Specifically, we will compare the performance of some patients with visual impairments in one specific crowding-sensitive task, identification of crowded letters. In all cases, stimuli were presented in the center of the visual field as a function of spacing to show restored recognition. All patients have different etiologies with presumably diverse neural signatures.

In the second study, we will loosen the defining criteria to include both foveal and peripheral vision. In particular, we will present the recognition range restoration in impaired patients and unimpaired subjects tested in their peripheral vision using the same crowding-sensitive task. Although in this case, the condition requirements are more general, the neural substrates are clearly defined as being different based on the retinotopic mapping of foveal and peripheral stimuli.

#### Study 1: Foveal Crowding in Visually Impaired Patients

Here, we will consider the property of recognizing an object ***o*** placed between two other objects ***a*** and ***b*if**, and **only if**:

1. The object ***o*** is presented in foveal vision (same input location across all observers);
2. The objects’ size is greater than 0.02 degrees of angle (normal foveal visual acuity);
3. The distance between ***o*** and ***a***, ***o***, and ***b*** needed to restore recognition is greater than 0.07 degrees of angle (the range of normal foveal crowding).

To minimize the effect of crowding, we move the eyes and recognize objects using a retinal foveal region having a diameter of 2 deg (Wandell, 1995). In the first clause, we restrain the analysis only to foveal vision. This region has the highest visual acuity correlated with the smallest scatter and size of the receptive fields of ganglion cells (Hubel and Wiesel, 1974).

In the fovea, acuity (i.e., blur) may impair recognition independently of crowding (Song et al., 2014). Thus, the second clause excludes all the cases in which the limitations on visual recognition depend on visual acuity.

We are constantly surrounded by complex and cluttered scenes, and recognition requires a certain range of critical spacing between two objects (Manassi and Whitney, 2018). Visual crowding has a large critical-spacing range in the periphery and a small one in the fovea, measurable only when the objects are close to acuity (Flom et al., 1963b; Pelli and Rosen, 2015; Coates et al., 2018). So, the third clause is about the critical spacing needed to recognize the target in the fovea when presented in clutter, and it poses an important constraint for defining the cases that will be presented. Specifically, according to the defining criterion, in the patients, we expect recognition to be restored in the fovea with a critical spacing larger than normal, independently from etiology and neural loss (critical spacing >0.07 degrees, Pelli et al., 2016).

#### Study 2: Foveal Crowding in Visually Impaired Patients and Peripheral Crowding in Unimpaired Subjects

In this second example of multiple realization, we will consider the property of recognizing an object ***o*** placed between two other objects ***a*** and ***b*if**:

1. The objects’ size is greater than 0.02 degrees of angle (normal foveal visual acuity);
2. The distance between ***o*** and ***a***, ***o***, and ***b*** needed to restore recognition is greater than 0.07 degrees of angle (the range of normal foveal crowding).

This study presents some main differences compared to study 1. First, the deletion of clause 1 from study 1 entails the inclusion of both foveal and peripheral vision. We also turned the biconditional requirement applied in study 1 to a simple conditional statement because clause 2 implies a range greater than 0.07 deg to restore recognition. However, 0.07 deg would be a sufficient range for correct object identification in the normal fovea; thus, the condition in clause 2 is not necessary but only sufficient.

### Presentation of the Case Studies

#### Case 1

LM is a 71-year-old man and retired laboratory technician who suffered an ischemic stroke in the right posterior cortex at the age of 66 years that resulted in left homonymous hemianopsia (visual field loss on the left side of the vertical meridian) (Petersen et al., 2016; Sand et al., 2018).

LB is a retired academic man who suffered a bilateral stroke when he was 81 years old. He has also achromatopsia (color blindness) and topographical disorientation (deficit in navigating familiar external spaces).

Their critical spacing was measured with a recent test developed to study foveal crowding (Pelli et al., 2016). The stimuli were multiple repetitions of a random sample of two letters or digits covering the entire screen. Patients were required to report both while varying inter-item spacing. The spacing threshold (the minimum spacing between stimuli) was measured with an adaptive procedure to reach the 70% accuracy criterion level (see Pelli et al., 2016; Sand et al., 2018 for details). The two patients showed significantly more crowding than a control group (critical spacing *M* = 0.175 degrees of angle [deg], *SD* = 0.015 deg).

Both patients show cortical lesions after the posterior stroke, although with differences in the severity of the extension. For LM, magnetic resonance imaging (MRI) scans acquired 12 months after the stroke showed “an infarction located in the posterior right cerebral hemisphere. Anteriorly, the lesion extends into the right parahippocampal gyrus and, posteriorly, into the lingual gyrus and the medial part of the fusiform gyrus. The lateral portion of the fusiform gyrus is spared, but the white matter above it (the inferior longitudinal fasciculus) is affected. Medially, the lesion surrounds the calcarine sulcus from its most anterior to its most posterior part. There are also two small lacunar infarctions in the right thalamus as well as one in the right centrum semiovale (Sand et al., 2018). LB’s MRI scans showed bilateral occipito-temporal infarctions. The right hemisphere lesion extends from the anterior part of the parahippocampal gyrus and might also involve the posterior part of the corpus callosum. Posteriorly, the lesion extends into the lingual and fusiform gyri. In the left hemisphere, there is a small lesion located in the anterior part of the parahippocampal gyrus as well as a small lesion located close to the occipital pole. There are also bilateral white matter lesions in the parietal regions (see Sand et al., 2018 for the MRI images).

#### Case 2

Patient 1 is a 74-year-old housewife with a diagnosis of PCA, a variant of Alzheimer syndrome characterized by a gradual and progressive deterioration in visual perceptual skills.

Patient 2 is a 58-year-old former care assistant also showing a decline in several perceptual and cognitive tasks compatible with the PCA syndrome (Crutch and Warrington, 2007).

Their crowding range was measured with stimuli composed of target letters flanked by two letters at four spacing conditions (condensed, normal, two-space-expanded, and four-space-expanded). Both patients were severely impaired in this task compared with control subjects (critical spacing: patient 1, 1 deg; patient 2, 1.8 deg).

In patient 1, mild non-specific changes with preserved alpha rhythm were observed with an electroencephalography exam. The MRI scans showed a “mild generalized cerebral atrophy with slightly greater prominence of parietal convexity sulcal spaces.” Patient 2 showed an absence of alpha rhythm and extra slow activity in the right temporal region. Visual evoked potentials were normal. The MRI scans showed “mild sulcal widening around the calcarine fissure” (Crutch and Warrington, 2007).

#### Case 3

Six patients (age: *M* = 26.5, *SD* = 14.15) with amblyopia caused by an early onset of strabism were included in the study (Song et al., 2014). Amblyopia is a condition characterized by a decreased vision in an eye, the input of which is impairly processed by the brain, which over time favors the other eye.

The threshold spacing was measured in the amblyopic eye by varying the inter-letter spacing between a target letter and four flanked letters (above, right, below, and left) with an adaptive procedure to achieve the 50% of accuracy criterion level. All patients were more or less impaired in the crowding test (*M* = 1.21 deg; *SD* = 1.44 deg).

The neural substrates of these patients were not investigated in the referenced study (see section “Discussion” for more details about amblyopia and neural loss).

#### Case 4

BB was 72 years old at the time of testing. He turned bilaterally blind at the age of 10 years after a violent lime spill that burnt the anterior chamber of both eyes and the posterior of the left eye. Eyes have been sutured to prevent infections. He then studied in Braille and worked as a switchboard operator. At the age of 62 years, after 51 years of complete visual deprivation, he underwent an osteo-odonto-keratoprosthesis intervention to the right eye performed by Prof. Falcinelli at S. Camillo Hospital in Rome, Italy (Falcinelli, 1993). He recovered sight within a central visual field of 10 degrees and a normal foveal visual acuity of −0.04 logMAR corresponding to 0.07 degrees of visual angle tested with the Snellen eye chart. The visual field restriction is the consequence of the implant’s optical characteristics, and it is not due to retinal loss (Falcinelli, 1993). BB visual abilities have been extensively tested after 10 years of recovery (Martelli et al., 2000). He showed normal contrast sensitivity for static and moving gratings with a modest decay for all the spatial frequencies tested compatible with BB’s age and impairment in recognizing pictures of objects presented in an unusual perspective (Martelli et al., 2000). BB critical spacing was evaluated centrally measuring contrast threshold for a target letter as a function of the flankers’ spacing with an adaptive procedure converging at the 82% accuracy criterion. The critical spacing threshold is identified as the spacing at which letter recognition ability with the flankers equals the ability tested without flankers (i.e., the breakpoint of the function; Pelli et al., 2004). BB shows a large range of foveal crowding whereby recognition is restored if flankers are at a center-to-center spacing of 2.5 deg (Martelli, 2001). The neural substrate of the patient has not been investigated, and BB had no neurological history.

#### Cases 5

Three non-neurological university students with normal or corrected-to-normal acuity participated in this study (Pelli et al., 2004). Stimuli were presented binocularly at 4 deg of eccentricity, target and flankers size measured 0.32 deg. Apart from this difference, stimuli and procedures were identical to the one used in case 4. Data show that subjects required about 1.2 deg of inter-letter spacing (critical spacing) to restore recognition at the tested eccentricity (*M* = 1.28, *SD* = 0.19).

### Comments to Study 1: Foveal Crowding in Visually Impaired Patients

We presented four case studies of patients with visual impairments as explained by foveal crowding. Their visual impairment cannot be explained by early sensory deficits (e.g., low visual acuity or contrast sensitivity), oculomotor disturbances, attentional deficits, aphasic syndromes, and semantic dementia. Thus, referring to clause two and three, we took into account only those subjects whose visual impairment in object recognition might be explained by visual crowding and not acuity (for more details on the relation between visual crowding and acuity, see Song et al., 2014; Strappini et al., 2017b). These patients cannot correctly identify letters if presented foveally in a clutter. To investigate their crowding range, all patients were tested with comparable crowding-sensitive tasks that required the identification of a letter flanked by other letters. All patients obtained a critical spacing greater than 0.07 deg. Thus, patients show a dependency on spacing largely greater than the normal fovea to restore recognition. This sensitivity is a marker of the operation of integrating the visual elements necessary to restore the property of correctly identifying the object. The data show that this operation is realized in the same way in all the reported cases over and above the differences in the patients’ etiologies and neuroanatomical impairments.

### Comments to Study 2: Foveal Crowding in Visually Impaired Patients and Peripheral Crowding in Unimpaired Subjects

Overall, all the cases we presented are limited by crowding. Both foveal vision in visually impaired patients and peripheral vision in normal subjects require a critical spacing bigger than 0.07 deg to release from crowding and recover recognition. Although all patients presumably have a certain degree of neural impairment, the normal subjects are neurologically intact.

Study 2 identifies a precise correspondence between the critical spacing necessary to restore recognition for the impaired patients tested in foveal vision and the non-neurological observers in peripheral vision at an eccentricity in which the two estimates are equivalent. This comparison is crucial for our thesis because it is known that foveal and peripheral regions from the eyes project to different areas of the brain (see section “Discussion”). Thus, this result leads us to conclude that the psychological property of identifying an object is multiply realized by the foveal pathway in the visual brain of the impaired subjects and the visual peripheral pathway of the non-neurological subjects.

## Discussion

Despite several decades of research, the existence of empirical evidence in favor of intraspecific, human, multiple realization of mental processes and properties is still debated. In this study, we consider the psychological property of recognizing an object when presented in clutter a ubiquitous phenomenon of everyday life. In particular, we present two partially different evidence of multiple realization based on some cases of visually impaired patients and normal peripheral vision, both constrained by visual crowding. We show that despite all cases sharing an abnormal range of critical spacing to restore recognition, they highly differ in their neural systems.

In the next paragraphs, we will discuss whether these cases are all possible different realizations of the same psychological property. To that aim, we will first discuss the neural substrates of visual crowding in normal subjects and in the visually impaired patients.

It is crucial for empirical evidence of multiple realization that the neural realizers are at the same neural grain size level (Aizawa and Gillett, 2009a, b). The hierarchy of the nervous system is composed of many levels such as biological macromolecules, synapses, neurons, neural circuits, cortical areas, and systems of areas (e.g., visual system) (Liang et al., 2016, p. 14). In these studies, we will discuss the neural substrates at the level of cortical areas and networks. Here, we informally apply the term *network* to a set of areas that contribute to a particular set of tasks or functions without an explicit reference to the anatomical connections (Petersen and Sporns, 2015). This level has become the most common framework to describe the human cognitive architecture in the last decades (Raichle et al., 2001; Behrmann and Plaut, 2013; Petersen and Sporns, 2015).

### Neural Correlates of Crowding in Normal Subjects

It has been suggested that the site of visual integration involves several visual areas in the striate and extrastriate cortex. Most of the research about the neural substrates of the integration process comes from studies of visual crowding on normal subjects tested in peripheral vision. These studies generally agree on locating this phenomenon in the visual cortex beyond the site of binocular combination, based on the observation that there is visual crowding even when the target and the flankers are presented in dichoptic vision (Flom et al., 1963a). However, the precise locus or network is still debated. Some psychophysical and neuroimaging studies have suggested that V1 can be the earliest area showing neural activity modulated by crowding (Anderson et al., 2012; Millin et al., 2013; Chen et al., 2014; Kwon et al., 2014). However, crowding related activation in V1 is absent when attention is diverted away from the stimulus, indicating that V1 involvement in crowding may be the result of feedback suppression coming from higher-order areas (Strappini et al., 2017a).

Evidence for double dissociation of crowding and acuity (Song et al., 2014) suggests that acuity and crowding may be linked to different areas. Acuity is tightly linked to V1, so crowding may be tightly linked to a higher cortical network of regions (Song et al., 2014; Strappini et al., 2017b). Some studies have speculated that V2 has the critical receptive field size to induce crowding (Freeman and Simoncelli, 2011) and that its receptive fields, in synergy with spatial attention, modulate their size to reduce crowding (He et al., 2019). Others have pointed to V3 (Tyler and Likova, 2007; Bi et al., 2009), V4 (Motter, 2006), or higher visual areas (Chung et al., 2007; Freeman et al., 2011). Although the existence of a “crowding area” is still debated, the modulation of the neural activity from early to higher visual areas, like the visual word form area (VWFA), is consistent with the increase in the receptive field size (Freeman et al., 2011; Pelli and Rosen, 2015; Strappini et al., 2017a) and with the occurrence of visual crowding at multiple levels in the visual hierarchy (Whitney and Levi, 2011).

### Neural Correlates of Crowding in Visually Impaired Patients

In studying a psychological function, it is possible to obtain useful insights on its characteristics from the study of cases in which that function is impaired. If a neural structure plays a role in the realization of that function, then damage to that neural structure would lead to an impairment of the function.

The cases presented here show some examples of neural implementation of visual crowding. The first two cases, LM and LB (Petersen et al., 2016; Sand et al., 2018) suffered a stroke, a cerebral lesion in which the neuronal death depends on a sudden lack of adequate amount of blood flow, thus oxygen and glucose. Both patients show cortical lesions in the posterior part of the brain, including the visual cortex, specifically in the lingual and fusiform gyri (occipital and temporal lobe, respectively).

The second cases, conversely, have a visual impairment with a gradual onset attributed to PCA syndrome, a progressive neuronal loss in the posterior part of the cerebral cortex. Neuroimaging studies have shown that PCA correlates with severe hypoperfusion in the lateral and medial parieto-occipito-temporal cortices (Kas et al., 2011; Crutch et al., 2017).

Finally, the patients with strabismic amblyopia and restored sight can both be considered as clinical cases of visual deprivation that silence the retinal input from the eye. This is due to suppressive mechanisms on the one end and lack of sensory information on the other. Regarding amblyopia, it is still unknown whether the impairment is due to a feed-forward dominance or feed-back selection of the fellow eye through top–down mechanisms that originate in the extrastriate cortex (Kiorpes and Daw, 2018). Nevertheless, dysfunction in V1 does not seem to be sufficient to explain the visual impairment in amblyopia (Kiorpes et al., 1998; Shooner et al., 2015), whereas the neural correlates of late-blind patients are still unknown.

Overall, the patients are neurologically different in that two show a large loss of the ventral cortex (Sand et al., 2018), PCA patients show general hypoperfusion of the posterior cortex (Crutch et al., 2017), and amblyopes and the visually deprived patient have no evident neuronal loss. In general, it is possible to speculate that in all these patients, visual crowding might reflect a limitation in the number of neurons devoted to foveal integration. Although a few studies have correlated retinal ganglion cell density (Kwon and Liu, 2019) and the number of cortical neurons (Strappini et al., 2017b) to the critical spacing, these clinical cases also point to differences in the way the neuronal decrease can lead to visual crowding.

### Foveal Crowding in Visually Impaired Patients as an Instance of Multiple Realization

We can speculate on how visual integration is realized in the visual system as evidence of multiple realization. We should consider whether the neural substrate responsible for recognition at large spacing is the same or different in all these patients. Due to the heterogeneity across all these cases, it is unlikely that an identical neural correlate supports the function. However, we cannot exclude that the recognition ability is supported using the residual functions of the same network.

The network hypothesis requires further consideration about the way in which a network may contribute to the realization of a psychological property in a multiple realization perspective. We will first establish that all the nodes in the network are probably necessary but not sufficient for the network working. If we consider these regions working as “critical” hubs (thus, damage in one area does damage to all the circuit), the neural substrates of the patients would eventually be the same and considered as an example of “merging” of realizers. Several studies have shown that many visual areas are engaged in the representation of multiple functions (cf. review of Behrmann and Plaut, 2013; Kay and Yeatman, 2017). A malfunction of all the network would cause a variety of visual deficits, beyond integration, that has not been observed in the literature (Strappini et al., 2017b). Thus, we can conclude that each node is necessary but not sufficient for the network working.

We have strong physiological evidence that all the visual areas perform different types of processing of the visual input, beyond visual integration, such as detection of elementary features, color and motion perception, and shape processing. Because these nodes are necessary but not sufficient for the network functions, we may speculate on how a partial compromise of those nodes might possibly affect visual integration while sparing the other functions of the nodes.

Just for the sake of speculation, consider the following hypothetical example: it is reasonable to conjecture that an area that detects motion needs integration fields to compute the delay among different events that occur inside those regions. The same computation could be “adapted” to the integration of static visual features, simply setting the interval between the events to zero. This example would again be an instance of multiple realization in that the different areas of a network contribute to computing different features to the realization of the same psychological property, integration. This perspective would be compatible with an even more stringent version of multiple realization that supposes that the realizers are “differently the same” (Shapiro, 2004; Polger and Shapiro, 2016), that is, the differences among the realizers must be relevant to the way the property is realized.

### Foveal Crowding in Visually Impaired Patients and Peripheral Crowding in Unimpaired Subjects as an Instance of Multiple Realization

The second study presents a broad comparison between foveal and peripheral crowding as evidence of MRT. The normal range of crowding scales with eccentricity. However, psychophysical models have clearly shown that the computation, integration, is the same across the visual field. This view of visual crowding as an indivisible and homogeneous phenomenon across the visual field contrasts with the high diversity of its neuroanatomical substrate across the foveal and peripheral vision. These neuroanatomical differences are remarkably relevant to MRT. Eccentric retinal regions project to the corresponding cortical areas that represent the peripheral parts of the visual field. This spatial specificity of connections between neurons contributes to the emergence of topographical cortical representations of the visual field (retinotopic maps). In the human primary visual cortex, as an object moves from foveal to peripheral locations of the visual field, the neurons that are activated varies from posterior to anterior parts of the calcarine fissure (Daniel and Whitteridge, 1961; Adams et al., 2007). This organization is preserved in the rest of the retinotopic visual areas (Wandell and Winawer, 2011). Although foveal and peripheral crowding may be associated with the same psychophysical mechanism, this topographical specificity hinders the hypothesis that they are based on the same anatomical structures.

Equating for the crowding range, here, we have shown that recognition is restored similarly in patients’ foveal vision as in non-neurological subjects tested in the periphery. Knowing that the neural structures recruited by foveal and peripheral stimuli are different, this is strong evidence for MRT.

### Contribution to the Debate on the Empirical Evidence of MR of Psychological Properties

In a paper on the multiple realization of psychological properties, Aizawa and Gillett (2009b) expressed the hope that more scholars would focus their attention on the multiple realization evidence coming from science. Indeed, they are firmly convinced that the discussion on MRT may turn from a traditional theoretical dimension, typical of philosophical debates, to a more concrete empirical evidence-based dimension. From this perspective, we hope that our proposal will contribute to the debate. Next, we will compare our contribution to three attempts to empirically test MRT reported in the literature.

#### Color Vision

Aizawa and Gillett (2009a) proposed an example of multiple realization based on normal color vision. Chromatic perception depends on the sensitivity to three different primary lights that are processed by three distinct retinal photoreceptors, the short (blue), medium (green), and long (red) wavelength cones. Their different spectral sensitivity is the result of the differences in the chromophore pigments, called opsins, that are contained in these cells. The authors noted that several studies have shown the existence of polymorphisms in the green and red opsins in the normal population. These small variations in the amino acid chains result in slightly different absorption spectra of the opsins, in particular for those codifying red and green. However, these slight variations are included in what is considered normal chromatic perception. Thus, the normal color vision can be considered multiple realized because there are normal variations in its parts.

In this example, the property levels are described in fine detail: the opsin properties multiply realize the photoreceptor properties related to the spectral absorption rate, which is relevant for the property of chromatic perception. However, it has been objected that these polymorphisms may be accounted for normal individual differences (Polger and Shapiro, 2016).

Somehow in line with Polger and Shapiro (2016), we think that in the example provided by Aizawa and Gillet, the cognitive property is missing. We do not exclude *a priori* that color perception (or being trichromat) can be considered a psychological property; however, we think that its phenomenology, its behavioral outcome, is missing from the proposal. We further conjecture that this example could provide concrete evidence of multiple realization if the psychological level was added by showing that there are no differences in color perception among trichromats that have those polymorphisms. Indeed, even slight differences among these normal trichromats would exclude that color vision is multiply realized.

In any case, even if the psychological property would be exactly the same across individuals, the reported evidence of multiple realization would concern low-level peripheral processing (proteins and biological macromolecules). The level at which MRT is usually considered as an alternative to the psychoneural identity theory is the neural level, including areas and networks of areas, which is the focus of our contribution.

#### Dendritic Spines

Aizawa and Gillett (2009b) discussed in detail another example of multiple realization of a psychological property, this time at the neuronal level. They start from the assumption that probably any psychological property depends in some way on the electrical activity of excitatory neurons across distinct regions of the cortex. This electrical activity may, in turn, be modulated and eventually multiply realized by the properties and relations of other parts of the neural structure. Neurons are notoriously composed of cell bodies, axons, and dendrites; through the activity of synaptic connections, the dendrites receive information from other neurons. The authors focused their attention on a particular substructure of the dendrites, known as dendritic spines. It is believed that dendritic spines may play a role in the memory storage, in the modulation of synaptic strength, and in the transmission of the electrical signal. Dendritic spines have several properties, such as size, length, and volume. It has been shown that such properties may vary over time, from hours to weeks. This neuronal property, that is, transmitting electrical signals to other neurons, seems to be multiply realized by the properties of the dendritic spines – as they vary along a time dimension. Consequently, a psychological property is multiply realized by the properties of the dendritic spines. The authors suggested “remembering something” as an example of a psychological property. Remembering something may remain constant in the same individual, whereas the properties of the dendritic spines vary in time as the properties at the neuronal level.

Although the description of the realizers is very detailed, as noted by the authors, we argue that it cannot be excluded that one day, it will be discovered that the plasticity of the dendritic spines actually does not play any relevant role in the realization of the psychological property, that is, remembering something.

The evidence we provide is not susceptible to the same objection in that the relationship between the psychological property we have described and the associated neural realizers is clearly defined.

#### Psychopathology

Finally, it is worth mentioning a seminal work recently published by Borsboom et al. (2019). Although the authors’ attention is focused on clinical psychology and what may be classified as pathological thoughts, believes, and behaviors, their framework can be applied to mental states in general as well. In the authors’ view, the reference to MRT is actually far beyond the limit of the brain and the way in which the neural states realize the psychological properties. Indeed, it extends to a complex network of interconnections between the subject’s intentional states (thoughts, desires, and beliefs), the neural states, and the surrounding environment. Briefly, an individual, particular, mental state (e.g., fear) is determined by a coherent pattern of interconnections between the subject’s behavior and the surrounding environment (e.g., the subject tries to hide). Generally, we tend to interpret such a pattern of interconnections by making reference to the subjects’ intentional contents (e.g., the subject believes that hiding will reduce the fear). Yet, this inference is not enough to understand the exact mental state of the subject and if the behavior is appropriate or dysfunctional. Indeed, the appropriateness of the subject’s behavior depends also on cultural and social factors. Consequently, according to the authors, mental states may be realized in many different ways in different people.

Although this framework is conceptually plausible and intriguing, it does not seem to provide compelling evidence in its favor. Let us consider how the desire of taking an umbrella may be realized at the brain level. Under the framework perspective, the desire of taking the umbrella cannot be isolated from a more complex network of related mental contents (e.g., the belief that it will probably rain; the belief that the umbrella will protect you from the rain or sun; the belief that it is not good to expose yourself to the rain or the sun, etc.). These contents may be extremely diverse and idiosyncratic. Thus, it is obviously very unlikely that, in our example, the desire of taking the umbrella would correspond to the same pattern of interconnected intentional states in different subjects, as for the neural realizers of such intentional states. Therefore, the theory simply states that it is highly improbable that the mental contents are realized by the same neural substrates in different subjects.

Here, the theory may be subjected to what has been termed the “Grain-Argument” objection (Bechtel and Mundale, 1999; Aizawa and Gillett, 2009b). According to this argument, although the “grain” at which a psychological property is usually described is coarse, the level at which the supposed neural substrates are described is much finer. As a consequence, a property described vaguely may actually be related to a variety of brain states individuated at a much finer grain. This reasoning may give rise to the illusory impression that the mental property may actually be realized by many different neural substrates. In line with this reasoning, it has been objected that in this framework, the mental properties can be subjected to kind splitting (Pernu, 2017). Reducing the grain at the psychological level may reduce the variability observed at the neuronal level and increase the possible correspondence between the psychological properties and neural realizers.

Although we consider this proposal as a cornerstone that will inspire future promising research attempts at testing MRT at an empirical level, comparing their network theory to our proposal, we may highlight two main differences: (1) our evidence refers more strictly to the relation between the psychological property and the neural realizers; (2) compared to the intentional states, whose nature and status is undoubtedly more complex, we refer to a simpler and indivisible psychological property (i.e., the ability to recognize an object as a function of a physical stimulus parameter spacing), which may turn out to be relevant in response to the Grain-Argument objection and taxonomic “kind splitting” (Polger and Shapiro, 2016).

### Is Our Proposal Question-Begging?

Various formulations/definitions of MR may be taken into account to verify whether a property may be multiply realized. Each definition establishes some criteria that should be fulfilled in order for some evidence to be considered a case of MR. To test whether our empirical evidence could be considered an example of MR of a psychological property, we chose the definition of MR proposed by Aizawa and Gillett (2009a). Compared with other formulations (e.g., Polger and Shapiro, 2016), it is not the most conservative. The comparison among different formulations of MR is surely an interesting issue to be discussed, but it is more theoretical/philosophical in itself and, as a consequence, beyond the limit of the present paper, which is mainly empirical. Under this respect, the fact that the Aizawa and Gillett definition of MR is less conservative does not make it trivial the quest for properties that could be multiply realized. Even if it would turn out that, in the end, everything is multiply realized (which is of course far from obvious), it would remain in any case a question of empirical evidence.

Whether or not it does exist a human intraspecific case that fulfills the conditions proposed by Aizawa and Gillett is an empirical issue, and our paper is in fact aimed at looking for an answer to such an empirical question.

## Conclusion

In this study, we considered the visual phenomenon of crowding in visually impaired patients and normal subjects as possible evidence of human multiple realization of mental properties. We further discussed the virtues and limits of our proposal compared with some previous empirical evidence reported in the literature.

Although we acknowledge that our evidence is far from conclusive, we think that it provides a fruitful bridge between philosophical and scientific approaches in the study of the relationship between mental properties and the human brain. In particular, we anticipate that our proposal, integrating findings from neuropsychology and psychophysics, will help brain scientists to search for hypothetical multiple realizers by considering the compatibility of their data with the multiple realization view. Consistently, it has been suggested that scientists might already have produced such data, although rarely does the term “realization” appear in their works (Aizawa and Gillett, 2009b). This type of data could be for example the presence of some relevant “outliers,” that rather than being a nuisance to regress out might indicate the existence of greater, unknown complexity in the studied phenomenon.

## Data Availability Statement

All datasets generated for this study are included in the article/supplementary material, further inquiries can be directed to the corresponding author.

## Author Contributions

EP had the idea to compare visual crowding and agnosia as a possible example of multiple realization. All the authors contributed to the conjecture and writing.

## Conflict of Interest

The authors declare that the research was conducted in the absence of any commercial or financial relationships that could be construed as a potential conflict of interest.
